# *Retracted*: Effects of major ingredients (grape syrup, milk powder, and walnut paste) levels on physiochemical, rheological, and sensory attributes of walnut spread

**DOI:** 10.1002/fsn3.3107

**Published:** 2022-10-20

**Authors:** Siamak Rahbari, Hamid Tavakolipour, Ahmad Kalbasi‐Ashtari

## Abstract

Effects of major ingredients (grape syrup, milk powder, and walnut paste) levels on physiochemical, rheological, and sensory attributes of walnut spread, *Food Science & Nutrition*, 2022 (https://onlinelibrary.wiley.com/doi/full/10.1002/fsn3.3107). The above article, published online on October 20, 2022 in Wiley Online Library (https://wileyonlinelibrary.com), has been retracted by agreement between the authors, the journal Editor in Chief Y. Martin Lo, and Wiley Periodicals LLC. The retraction has been agreed due to an error in which the incorrect manuscript was sent to the production team and published instead of the manuscript that the journal had reviewed and accepted.

[1]Department of Food Science and Technology, Tehran North Branch, Islamic Azad University, Tehran, Iran

[2]Department of Food Science and Technology, Sabzevar Branch, Islamic Azad University, Sabzevar, Iran

[3]Biological and Agricultural Engineering Department, Texas A&M University, College Station, Texas, USA

### Abstract

Separate levels of roasted walnut paste [illegible] 25, 30, and 3[illegible] Persian grape molasses (PGM) (40, 50, and 60 g), and high protein m[illegible] powder (HPMP) (5, 7.5, and 10 g) were grinded and refined in a b[illegible] for 3 h at 45[illegible]ater, the peroxide value (PV), $a_w$, acidity, color indexes, ac[illegible]eptable sensory score (ASS), texture, and rheological properties of the resulting w[illegible]nut spreads ([illegible]S) were determined. The response surface methodology and centra[illegible]mposite de[illegible]gn were used to find the optimized WS formulation, whic[illegible] 50 g PGM, [illegible]MP, 30 g RWP, and ASS = 91 of 100. The mathematical mod[illegible] of AS[illegible] good fitness's ($R^2 > 98\%$) with the ingredient's levels and could predict the [illegible]S[illegible] new WS without any sensory test for reassessment. The PV, a[illegible]ity of [illegible]imized WS did not change even after 90-day storage at 4°C. Wh[illegible]e the op[illegible]nized W[illegible] sample had very low stickiness (0.1–0.6 mJ), its hardness, ad[illegible]iven[illegible]ess, springiness, gumminess, and chewiness, respectively, decrea[illegible] 37%, 83%, 3%, 6%, 25%, and 29% when its temperature increased from 4 to 25°C. [illegible]e viscoelastic results showed that the RWP plays a dual role and acts [illegible] a filler in its complicated network of the WS product. Most probably significant int[illegible]ctions take place between crystallized HPMP and PGM to make hydrodynamic polymers with the oil base of walnut paste. While the walnut spread had a long shelf life at 4°C, it is a high protein food with considerable unsaturated fatty acids and antioxidants.

RETRACTED

### 1 | INTRODUCTION

Different milk spreads are produced by particle size reduction in different ingredients (paste of different nuts and specifically hazelnut, milk &/or cocoa powder, and cocoa butter) followed by the ball milling (Amevor et al., 2018; dos Santos et al., 2020). Traditionally, the ball mill equipped with a horizontal feed cylinder located on the top of 3–5 vertical stacked rollers. When the mixed materials passes mechanical or hydraulic rollers, all the crystal sugar and solid materials break to very fine particles (~30 um), which cannot be felt on the tongue (Amevor et al., 2018; Savitri et al., 2021). This is the main reason that many people like the excellent taste of milk spread because it is melted on their tongue. Additionally, the gradual movement of mixed ingredients on the sequential rollers remove their undesirable flavors and develop their pleasant aroma in the final spread. In other words, the heterogeneous, flaky, and relatively dried paste of each ingredient is transformed into a free-flowing suspension of solid particles dispersed in a dominant phase of used fat (Acan et al., 2021). Milk spreads are classified as dark, white, or chocolate, depends on the major ingredients used in their manufacturing. The inclusion of 10%–20% milk powder in any kind of nut-spread affects its physiochemical, rheological, and sensory characteristics (Monteiro et al., 2018). Most frequently, the milk powder (with or without fat) used in spread production is made by either roller or spray drier (Coutinho et al., 2019; Crawford & Running, 2020).

Whereas crystal sugar is used in most of different spreads (as a sweetening agent), it was our interest to substitute it the Persian grape molasses (PGM). The PGM with ~76% monosaccharide sugars (mainly glucose and fructose), minerals, organic acid and considerable bioactive compounds is a sweet liquid that is made from grape or raisin (Tavakolipour et al., 2020). Since PGM has interesting rheological properties and organoleptic (texture, color, and flavor) specifications, it will enhance the quality aspects of different products, if is used as an ingredient (Azizi Tabrizzad, 2020).

It was our objectives to combine roasted walnut paste (RV[illegible]), high protein milk powder (HPMP), and Persian grape molass[illegible] (PGM) each one at three levels and apply ball mill f[illegible]king various kinds of walnut spreads. Then study the[illegible]chemic[illegible] physical, rheological, and sensory characteristics [illegible]uring stor[illegible]e. Later find out the best levels of ingredients f[illegible] ma[illegible]g a [illegible]vel walnu[illegible] spread.

Linseeds and flaxseeds nutriti[illegible]are similar; th[illegible]nly difference can be seen in the plant it[illegible]f. Whi[illegible]he people in the UK and Australia distinguish betwe[illegible] linseed and fl[illegible]in the United States and Canada, they refer t[illegible]both as flax.

While the world[illegible]roduction[illegible] walnut was 4.5 million tons in 2019, the major prod[illegible]rs (i[illegible]e order of decreasing harvest) were China, the Uni[illegible] States, [illegible]n, and Tur[illegible] (FAOSTAT, 2019). The botanical st[illegible]ture [illegible] walnut ([illegible]lan[illegible]esia) is compared with human brain. [illegible]it is consi[illegible]red as the br[illegible]n nutrient (An et al., 2020). Walnut is rich [illegible]mono[illegible]ty acids (Omega 3), arachidonic acid, phyto-che[illegible]al substances, and bio-actives (melatonin, ellagic acid, vitamin E, ca[illegible]noids, and polyphenols). These compounds have potential health [illegible]ects against: aging, cancers, inflammations, and neurologic illnesses (Şen & Karadeniz, 2015).

Additionally, the 1 oz of walnut meal obtained after cold press provides 2.6 g of Omega-3 antioxidants. Linseed cake or meal resulted from cold pressing of its seeds has 40% protein, 6% minerals (ash), and 12% oil it is the most effective material for roasting and making linseed paste with interesting sensory attributes (Farag et al. 2021). The insoluble dietary fiber component of linseed (~7%) is effective in constipation relief, colon health and may even protect against colon cancer (Condori et al. 2020).

Different milk spreads are produced using most of the times in ball milling system to reduce particle size of diverse ingredients (different nuts, milk &/or cocoa powder, and cocoa butter) (Amevor et al., 2018).

Many people like the excellent taste of milk and nuts spreads because they are melted on their tongue due to their fine particles. Additionally, the gradual breaking of mixed ingredients removes the undesirable flavors of used ingredients and devel[illegible]leasant aroma in the final spread. In other words, the heteroge[illegible]ous, flaky, [illegible] relatively dried paste of each ingredient in LS prod[illegible]is transformed i[illegible] a free-flowing of solid and fine particles dispersed [illegible] dominant fat [illegible]hase of spreads (Acan et al., 2021). The i[illegible]usion of 10[illegible]20% milk [illegible]owder in any kind of nut-spread affects [illegible] physioch[illegible]mical, r[illegible]al, and sensory characteristics (Mont[illegible] et al., 2[illegible]). Furthermore, the method of milk drying (roller or [illegible]ray d[illegible]ion) for its [illegible]nversion to powder has effects on sen[illegible]y attributes [illegible]nal spr[illegible]d when it is used as an ingredient (Co[illegible]al., 2019; Craw[illegible]& Running, 2020).

Since there is tren[illegible] reduce crystal sugar (as a sweetening agent) i[illegible]st of food pr[illegible]cts, it was our interest to substitute cry[illegible] sugar with the native and local Persian grape molasses (P[illegible]M). While PGM has more than 50% sugar (mainly glucose and fr[illegible]ose) and depe[illegible]s on its Brix, it has considerable minerals, organ[illegible]cid, bioacti[illegible] compounds that is made from grape or raisin (Azizi Ta[illegible]020). Since PGM has interesting rheological prop[illegible]s and organoleptic (texture, color, and flavor) specifications, [illegible] will [illegible]ance the quality aspects of different products, if is used properly as an ingredient in food product (Azizi Tabrizzad, 2020).

It was our objectives to study the effects of mixing different levels of three valuable ingredients including roasted linseed paste (RLP), high protein milk powder (HPMP), and Persian grape molasses (PGM) on the physiochemical and sensory attributes of resultant linseed spreads (LS). Then evaluate the peroxide value (as a most important deteriorative index) of LS during long time (90 days) cold (4°C) storage. Then apply respond surface method and central composite design on the generated data and find out the best levels of mixing ingredients for making a highly nutritional and novel linseed spread with minimum deteriorating indexes. Then evaluate the physical, rheological, and sensory characteristics of optimized sample of LS before and after storage.

### 2 | MATERIALS AND METHODS

#### 2.1 | Materials

Grape seedless and walnut were prepared from local markets in Miandoab, West Azerbaijan Province, Iran. Skim milk powder was prepared from Pegah company of Tehran, Iran, and milk protein concentrate of 85% was prepared from Alinda company, Greece. Liquid lecithin was prepared from Behpak company, Behshahr Mazandaran Province, Iran. Wheat flour was provided from Taravat flour company, Miandoab, West Azerbaijan Province, Iran. The chemical compounds were purchased from Merck Corporation (Germany).

#### 2.2 | Preparation of high protein milk powder

The high protein milk powder produced by mixing Skim milk powder and milk protein concentrate (1:1) and the uniform powder mixture was packed in sterile plastic bags until consumption.

#### 2.3 | Preparation of Persian grape molasses

First, grapes seedless arrived at Nikan Pajouhan-Bonab laboratory, the leaves and seeds were separated and grape juice was prepared by a press, and grape juice was extracted by a water intake device. The pH, acidity, and brix of grape juice were measured. To reduce the acidity and remove the turbidity of grape juice, bentonite (3 g per 100 ml of grape juice) was used. The sap soil is first dissolved in a quantity of grape juice, and then the production treatment is added and thoroughly mixed. After about 3 h and at the end of the effect of the clarifying compounds and the help of the filter, the surface of the juice is cracked, at which time the existing foam must be removed from the surface of the juice and smoothed by a suitable filter. In the next step, grape juice with a certain amount of calcium carbonate is neutralized until it reaches a pH of 5.6. After neutralization, sap soil was used to remove turbidity. Finally, after 30 min and filtration, the production sample was subjected to vacuum cooking and [illegible]centration in the evaporator to a degree of 69 ± 1 brix (Tavakolip[illegible] et al., 2020). Production samples (ash 1.75%, pH 5.6, acidity 0.162[illegible] OA) were packaged and stored in sterile containers u[illegible] test.

### 3 | PREPARATION METHODS, MEASURING SPECIFICAT[illegible]NS, A[illegible] MODELING

#### 3.1 | Preparation a[illegible] processing

The walnut paste ([illegible] 3.14%, [illegible]tein 21.7%, fiber 18.22%, fat 37.86%, and pH 6) was [illegible]ade [illegible]oasting its seeds at 121°C for 1 min followed by its [illegible]niform [illegible]ng with~ [illegible]% lecithin (as emulsifier) at 45°C fo[illegible]0 min [illegible]urner & [illegible], 2011). Then, the null-flour of whe[illegible] roasted f[illegible] 140 s at 18[illegible]C (Germishuys et al., 2020) and mixed [illegible]te of [illegible]IP. Later, the PGM was added evenly to the pre[illegible]d mixture, and the contents was stirred for 10 min at 25°C to dev[illegible]p a liquor phase (Turner & McNiven, 2011). The production of wal[illegible] spread was completed using a ball mill (Sepehr machine, Iran) equipped with an agitator (600 RPM) containing 6 mm diameter stainless-steel balls using the technique of Bolenz et al. (2014). The mass of ultimate mixture was flowing through the balls (as a bed) using a recycling pump at medium current of 1 kg/min. The temperature of refining (particle size reduction) process was stabilized at 45°C and checked every 5 min during 3-h milling time. If the temperature of mixed materials goes above 45°C, the vessel was cooled with tap water before proceeding. A micrometer caliper was used to keep track of particle size until it reached 30 μm. With this setup, there was no way to reduce the size anymore. To prevent contamination, the molded product was left out for 3 h and wrapped in aluminum foil (Bolenz et al., 2014).

#### 3.2 | Chemical characterization

The following tests were carried out to measure [illegible]hemical specifications of LS:

a. Total dietary fiber using the enzymatic g[illegible]metric assay, b[illegible]ed on the AOAC-991.43 and AACC[illegible].07.01 me[illegible]d. Simila[illegible], crude protein measurement was [illegible]arried out by AAC[illegible]30 using a correction index of 5.9[illegible] nitrog[illegible]o-protein conversion.
b. Fat content utilizi[illegible] Sox[illegible]tractor (V[illegible], Italy) on 5 g of ground sample [illegible]ed with hex[illegible] as sol[illegible]t.
c. Ash content [illegible]m[illegible]ral residue by [illegible]ration of its total organic matter in a muffle fu[illegible]ce at 550°C, as described in the official AOA[illegible]rnational met[illegible] (AACC, 2000; AOAC, 2000).
d. W[illegible]er activity by direct met[illegible]od at 25°C through the $a_w$ analyzer [illegible]ovasina AG, S[illegible]tzerland).
e. [illegible]ee fatty acids (a[illegible]ree oleic acid percentage) and peroxide value b[illegible]plying the [illegible]CS methods (AOCS, 1989).
f. pH w[illegible]ed using a pH Meter (IKA, Germany) which was [illegible]librated before the test.

#### 3.3 | Sensory evaluation

After selecting 20 members of sensory panel (from the Department of Food Science and Faculty in the University of Tehran), they were trained for sensory evaluation. The evaluators assessed 8–10 g of prepared WS samples in nibble forms and in separate areas with air circulation under bright light (recommended by ISO Standards No. 8589 [2007]) 2 h after breakfast (ISO, 2007). We used a 5-point hedonic scale to score different sensory attributes (appearance, aroma, sweetness, mouthfeel, and aftertaste) of each sample (Mahato et al., 2021). The final sensory acceptance score of the samples is calculated by summing the average scores of five different sensory characteristics obtained from the evaluators. Although this approach does not represent accurate customer perception, but it strongly verified the required characteristics that the high-quality WS product should have it.

#### 3.4 | Experimental design, model fitting, and variables optimization

Central composite design (CCD) system was used to study the effects of three (PGM, HPMP, and RWP) independent parameters on the peroxide value, $a_w$, acidity, and sensory scores of the resulting samples of LS. The design included 20 experiments, which consisted of six center points in a cube. The operating conditions were conducted

RETRACTED

at five levels coded as −2 (−α), −1, 0, +1 and +2 (+α). The actual and coded values of the independent variables are listed in Table 1.

A second-order polynomial model expressed the relationship between the three independent variables and their responses for dependent variable (α):

$$\alpha = \beta_0 + \sum_{i=1}^{k} \beta_i x_i + \sum_{j=i+1}^{k} \beta_j x_j + \sum_{l=j+1}^{k} \beta_l x_l + \sum_{i=1}^{k} \sum_{j=i+1}^{k} \beta_{ij} x_i x_j + \sum_{i=1}^{k} \sum_{l=j+1}^{k} \beta_{il} x_i x_l + \sum_{j=i+1}^{k} \sum_{l=j+1}^{k} \beta_{jl} x_j x_l + \sum_{i=1}^{k} \beta_{ii} x_i^2 + \sum_{j=i+1}^{k} \beta_{jj} x_j^2 + \sum_{l=j+1}^{k} \beta_{ll} x_l^2 \tag{1}$$

Where **α** represents the response variable, $\beta_0$ is the constant coefficient, x represents the independent variable (factor), i is the $i^{th}$ factor, $\beta_i$ $\beta_j$ $\beta_l$, $\beta_{ii}$ $\beta_{jj}$ $\beta_{ll}$ and $\beta_{ij}$ $\beta_{il}$ $\beta_{jl}$ are the linear, quadratic, and second-order interaction coefficients, respectively. The statistical software Design-Expert®, version 11, was used for experimental design, analysis, graphing, and optimization.

Validation of the optimized conditions was carried out in triplicate to generate the optimum model using Duncan's new multiple range test (MRT). MRT is a multiple comparison procedure developed by David B. Duncan in 1955. The significance level used for this study was 95% ($p < .05$) for all the statistical analyses.

### 3.5 | Physical specifications (color values and texture profile analysis)

The surface color of optimized WS samples was [illegible]easure[illegible]n triplicate on 0, 45 and 90 days of storage. The CI[illegible]ab color c[illegible]rdinates including L (lightness), a (redness to gree[illegible]ness[illegible]nd [illegible]yellowne[illegible] to blueness) were determined using [illegible]roma Me[illegible] (Model D65, Minolta Co., Ltd.). Additionally, it w[illegible]librated using a [illegible]inolta calibration plate (Y = 94.75, x = 0.2[illegible] y = 0.4[illegible] The following equations were used to determine th[illegible]chroma, whitis[illegible]WI), yellowness (YI), and brownness (BI) inde[illegible]s of samples, based o[illegible]olor coordinates (Ekrami et al., 2019; [illegible]olková e[illegible]., 2021):

$$\text{[illegible]na} = \sqrt{a^2 \text{ [illegible] } b^2} \tag{2}$$

$$\text{[illegible]} = 100 - \sqrt{(100 - L)^2 + a^2 + b^2} \tag{3}$$

$$\text{YI} = \frac{142.86 \times b}{L} \tag{4}$$

$$\text{BI} = \frac{100 \times (X - 0.31)}{0.17}\ \ X = \frac{(a + (1.75 \times L))}{(5.645 \times L) + a - (3.012 \times b)} \tag{5}$$

Texture of each WS sample was measu[illegible]ith a Texture Analyzer (Stable Micro System Ltd., UK), an[illegible]cording t[illegible]ightingale et al (Nightingale et al., 2011) method [illegible]h some modifi[illegible]ion. An aluminum cylinder probe with 50 mm dia[illegible]er attached t[illegible] 0.5 N compression load. Three speeds [illegible] 2.0 (as a p[illegible]test), 1.0 [illegible]s a test), and 2.0 mm/s (as a post-test[illegible]ere used [illegible]n the 1[illegible]3[illegible]st samples, and the instrument was a[illegible]ted to [illegible]ieve 25% compression with 5-s waiting periods. T[illegible] textu[illegible]es were a[illegible]yzed for hardness, cohesiveness, spr[illegible]iness, adhe[illegible]ness, [illegible]mminess, and chewiness. The att[illegible]ute[illegible]f hardness an[illegible]ewiness were selected as essential response varia[illegible]s of final LS.

### 3[illegible] | Rheolo[illegible]ical description

The [illegible]eological pr[illegible]erties of the prepared WS were determined using a [illegible]in and temperature-controlled rheometer (Anton [illegible] GmbH, Austria) equipped with parallel plates geometry (each [illegible]e wi[illegible]50 mm diameter), and according to Mantihal et al. [23] method, with some modification. In the first, the gap between the two plates of rheometer was adjusted to 0.5 mm. After loading samples, its probe was descended and compressed the two plates with a gap of 2 mm. Then, viscosity measurement was started after 2 min for equilibration of the temperature and relaxation of the samples. The applied shear rate range was between 1 and 100 $s^{-1}$ radian frequency at 25°C. A power-law model (Fanari et al., 2020) used to show the dependency of the measured complex viscosity data on the frequency of LS.

$$\sigma = \boldsymbol{K}(\gamma)^{\boldsymbol{n}} \tag{6}$$

where σ is shear stress (Pa), K is the consistency coefficient (Pa·$s^n$), γ is the shear rate ($s^{-1}$), and n is the flow behavior index.

**TABLE 1** [illegible]tual and coded independent v[illegible]bles employed in experimental d[illegible]gn to find the specifications of optimized linseed spread after using response surface methodology

| Parameters | Coded value −2 (−α)[a] | −1 | 0 | +1 | +2 |
|---|---|---|---|---|---|
| PGM (Persian grape molasses) in (g) | 30 | 40 | 50 | 60 | 70 |
| HPMP (high protein milk powder) | 1 | 3.75 | 6.5 | 9.25 | 12 |
| RLP (roasted linseed paste) | 7.5 | 15 | 22.5 | 30 | 37.5 |

[a]Although only three levels of each ingredient were used to produce the LS with highest sensory scores and permissible deteriorative indexes (PV, $a_w$, and acidity), the response surface method (RSM) chose extra upper and extra lower level (in addition to its 3 levels) for each ingredient to find the optimized formula for making linseed spread with highest quality (sensory score) in spite of the chosen levels for each ingredient. α → K = 2.

RETRACTED

RETRACTED

## 4 | RESULT AND DISCUSSION

### 4.1 | Chemical characterization (peroxide value, water activity [$a_w$], and acidity)

The peroxide value (PV) is still the most important chemical index for determining how quickly the walnut spread oxidize and influences its shelf life due to its oil portion and to some extent its moisture content. When the percentage of PGM, HPMP, and RWP in formulation of final WS; its PV changed from 0.51 to 1.70 meq $O_2$/kg when it stored at 4°C On 1st day of production. Additionally, the effects of three ingredients on peroxide formation of WS (shown in Figure 1) confirmed that the contents of RWP and PGM had more effects than HPMP on increasing this deteriorative index.

Beside controlling PV, it was our intension to select appropriate ingredients along with suitable processes to minimize $a_w$ in the final WS and protect it from microorganisms' activities. When the proportions of PGM and RWP changed, its $a_w$ changed from 0.51 to 0.76 in the final WS at 4°C On 1st day of production. Additionally, the effects of three ingredients on water activity of walnut spread (shown in Figure 2) confirmed that the contents of PGM and RWP had impacts on increasing this index ($a_w$).

Figure 3 shows the results of measuring free acidity (as a [illegible]centage of oleic acid/100 g) of the final LS. When the percentage [illegible] PGM, HPMP, and RWP in formulation of WS, its acidity increase[illegible] from 0.079 to 0.189 %OA when it stored at 4°C On [illegible] of production. In addition, this figure shows that the [illegible]her co[illegible]nts of RWP and HPMP resulted an increasing effec[illegible]n acidity [illegible] walnut spread; however, PGM had a decreasing in[illegible]uenc[illegible]

### 4.2 | Sensory evaluati[illegible]

When nutritive edible in[illegible]dients are processed [illegible]perly and combine with safety and [illegible]alth conc[illegible]s, there is a good chance to receive good consume[illegible]espo[illegible]es. Becau[illegible]e customer opinion is a practical qualit[illegible]evel eva[illegible]ion. This i[illegible]he reason profiling a new food prod[illegible] using [illegible]trained [illegible]nel[illegible]judgment is necessary to certify co[illegible]ctness an[illegible]eproducing [illegible] with uniform sensory attributes. Despit[illegible]e gro[illegible]g p[illegible] of high-quality cocoa and chocolate products, [illegible]ture is limited on the sensory attributes of different spreads.

The total sens[illegible] score, which obtained the acceptance levels in walnut spread, changed from 45.3 to 90.9 (depends on the proportion of PGM, HPMP, and RWP used in the resulting LS).

As Figure 4 shows, increasing the amount of the three major ingredients to some extent improved the sensory evaluation score of walnut spread. However, the negative taste of RWP was noticed when it increased >30 g in formulation. The higher content of RWP in the final WS provided more unsaturated fatty acids for oxidation, and therefore, more taste of rancid fat (bitterness) was recognized.

### 4.3 | Model fitting

Table 2 shows the effects of different levels of major ingredients (PGM, HPMP, and RWP) on sensory acceptance, PV, $a_w$, acidity and acceptance scores of final samples of walnut spread.

The independent (RWP, GS, and HPMP) and dependent (PV, aw, acidity, and sensory score) variables were fitted to the second-order model equation and examined for their goodnes[illegible] (Table 3). The analyses of variance were performed to det[illegible]ine the la[illegible] of fit and the significance of independent variabl[illegible]on linear (first o[illegible]r) and interaction effects along with quadratic (s[illegible]nd order) imp[illegible]cts on dependent variables (specificatio[illegible] of WS.

The regression results sh[illegible]ed that d[illegible]fferent [illegible] tested for all the response variables [illegible]re highl[illegible]dequate because they had satisfactory levels of [illegible](>80[illegible] that ther[illegible]vas no significant lack of fit in all the[illegible]esponse varia[illegible]s. The[illegible]gression coefficients are shown in T[illegible]e [illegible]nd the equati[illegible]or each of the response variables cou[illegible] be deri[illegible] from the predicted values of each response v[illegible]ble. The $R^2$ val[illegible] of all the responses exceeded 80%, indic[illegible]ing a high proportion of [illegible]ariability (as explained by the data). Th[illegible]efore, the resp[illegible]se surface models developed were adequate.

[illegible]e empirical m[illegible]el was developed by fitting the experimental d[illegible]obtained fr[illegible] CCD design into a second-order polynomial mathema[illegible]tion (Equation 1). The model could be fitted to the [illegible]ing second-order polynomial relationships (Equations 7–10):

$$\begin{aligned}Y_{\text{Peroxide value}} = &+1.58159 - 0.010494(\text{PGM}) - 0.031386(\text{HPMP}) \\ &- 0.027261(\text{RWP}) + 0.0015(\text{PGM})(\text{HPMP}) \\ &- 0.0022(\text{HPMP})(\text{RWP}) - 0.000194(\text{PGM})^2 \\ &+ 0.002709(\text{HPMP})^2 + 0.002827(\text{RWP})^2\end{aligned} \tag{7}$$

$$\begin{aligned}Y_{aw} = &+1.41193 - 0.0122397(\text{PGM}) - -0.026773(\text{HPMP}) \\ &- 0.024023(\text{RWP}) + 0.00065(\text{PGM})(\text{RWP}) \\ &+ 0.0008(\text{HPMP})(\text{RWP}) + 0.000909(\text{HPMP})^2 - 0.000473(\text{RWP})^2\end{aligned} \tag{8}$$

$$\begin{aligned}Y_{\text{acidity}} = &+0.35517 - 0.004441(\text{PGM}) - 0.012018(\text{HPMP}) \\ &- 0.010993(\text{RWP}) + 0.00019(\text{PGM})(\text{HPMP}) + 0.00002(\text{PGM})^2 \\ &+ 0.000818(\text{HPMP})^2 - 0.000395(\text{RWP})^2\end{aligned} \tag{9}$$

$$\begin{aligned}Y_{\text{Acceptance}} = &-401.57727 + 6.56955(\text{PGM}) + 10.69591(\text{HPMP}) \\ &+ 16.60841(\text{RWP}) + 0.04(\text{PGM})(\text{RWP}) + 0.184(\text{HPMP})(\text{RWP}) \\ &- 0.06417(\text{PGM})^2 - 0.812727(\text{HPMP})^2 - 0.319682(\text{RWP})^2\end{aligned} \tag{10}$$

To investigate the relationship between process variables and response variables from the developed mathematical model equations, 3D contour plots were constructed between two independent variables while keeping the 3rd variable constant. The intercept values for model of peroxide values (Equation 7) confirmed that the increasing and decreasing effects of, respectively, two ingredients (RWP and HPMP) and one ingredient of PGM on this index (see

RETRACTED

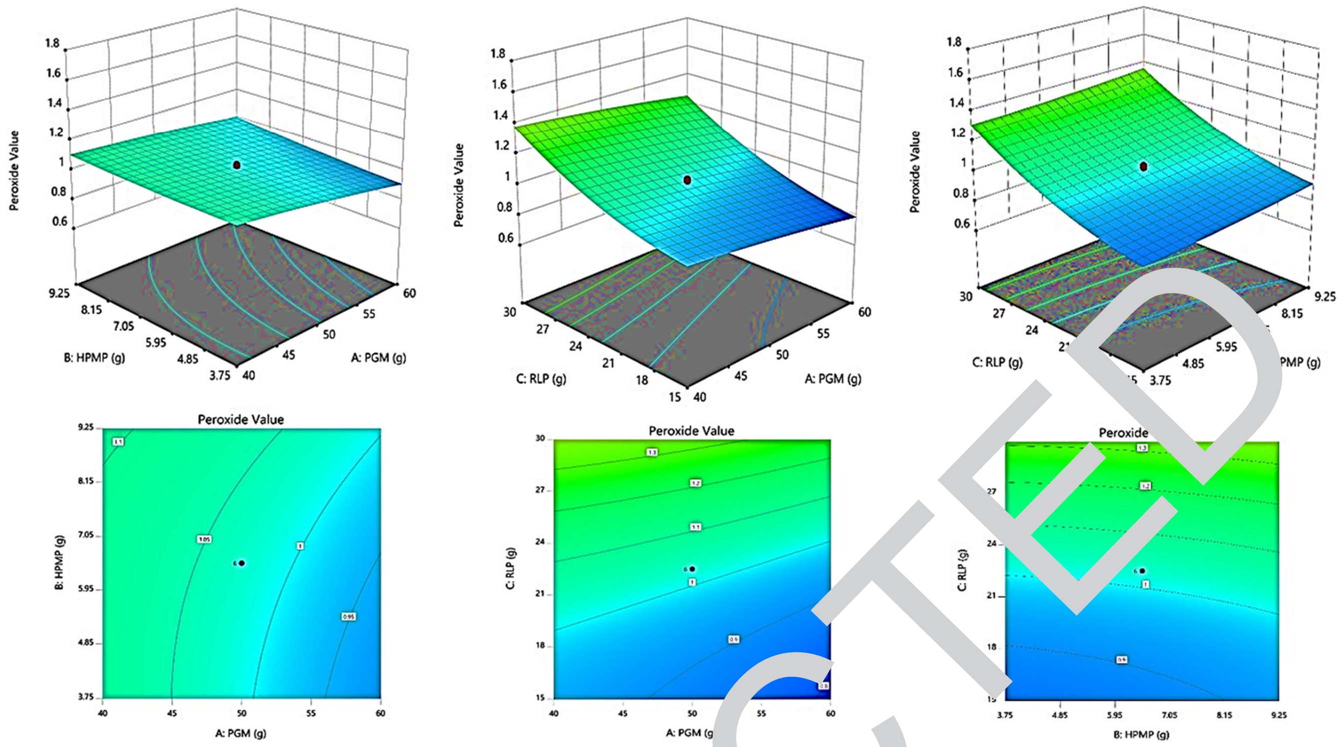

FIGURE 1 Response surface plots showing the interaction effects of independent variables of PGM, HPMP, and RLP (each one with five levels) on the peroxide value (PV) of the resulting LS samples after 90-day storage at 4°C

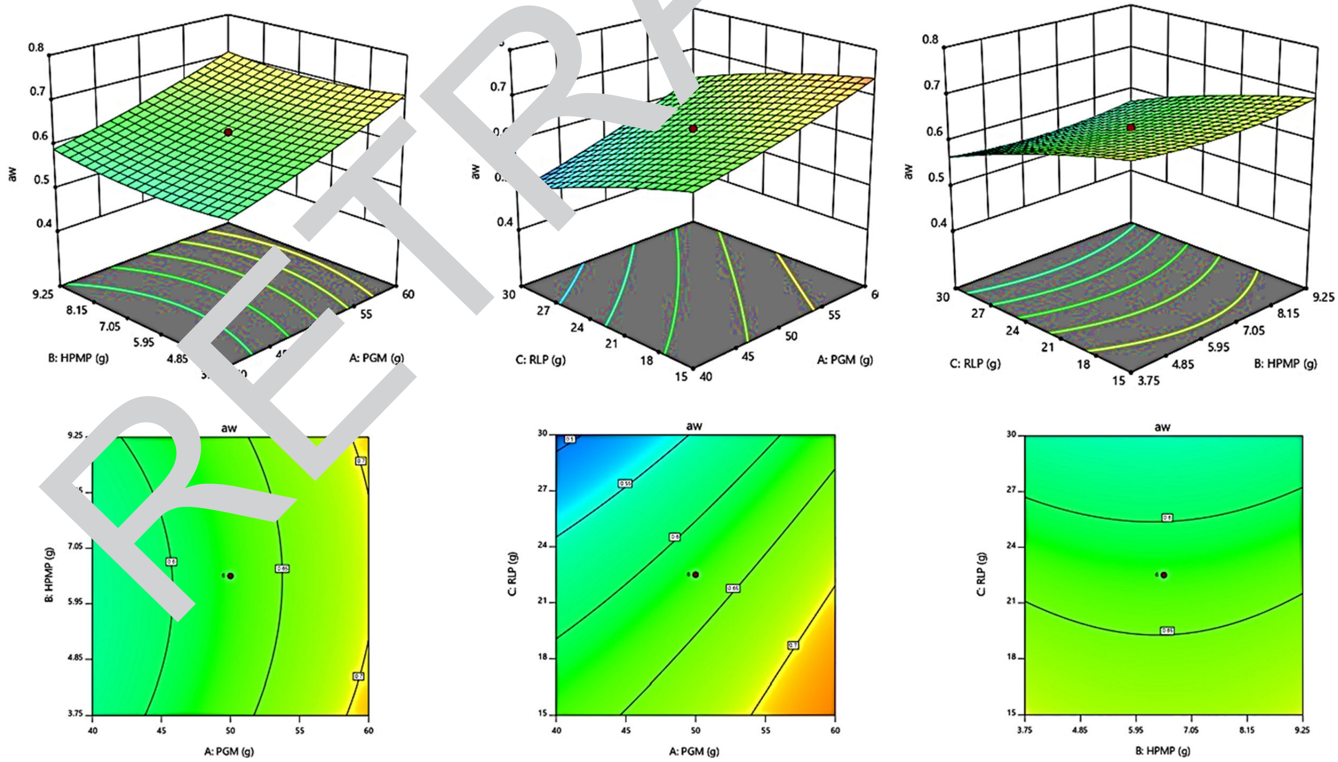

FIGURE 2 Response surface plots showing the interaction impact of independent variables (PGM, HPMP, and RLP) on the $a_w$ with different formulations of LS samples right after production.

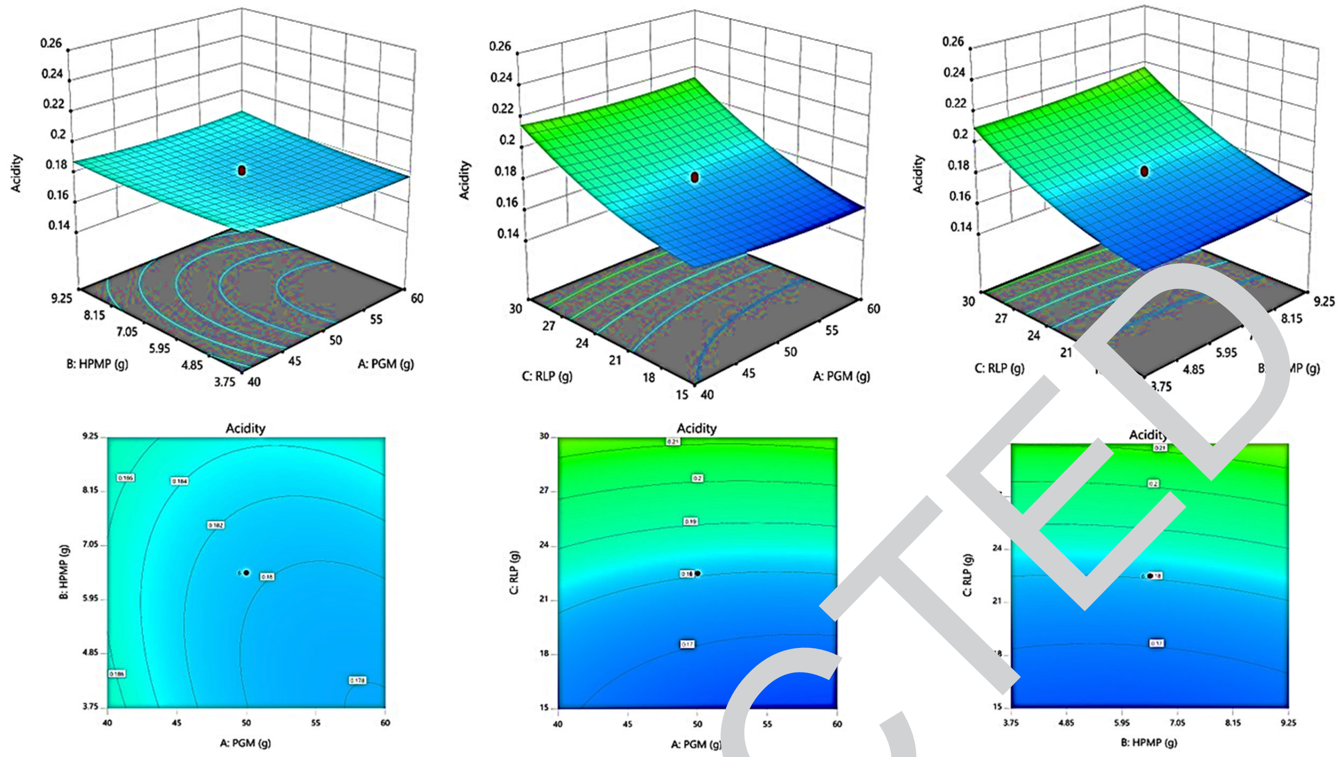

FIGURE 3 Response surface plots showing the interaction impact of independent variables (PGM, HPMP, and RLP) on the acidity with different formulations of LS samples right after production

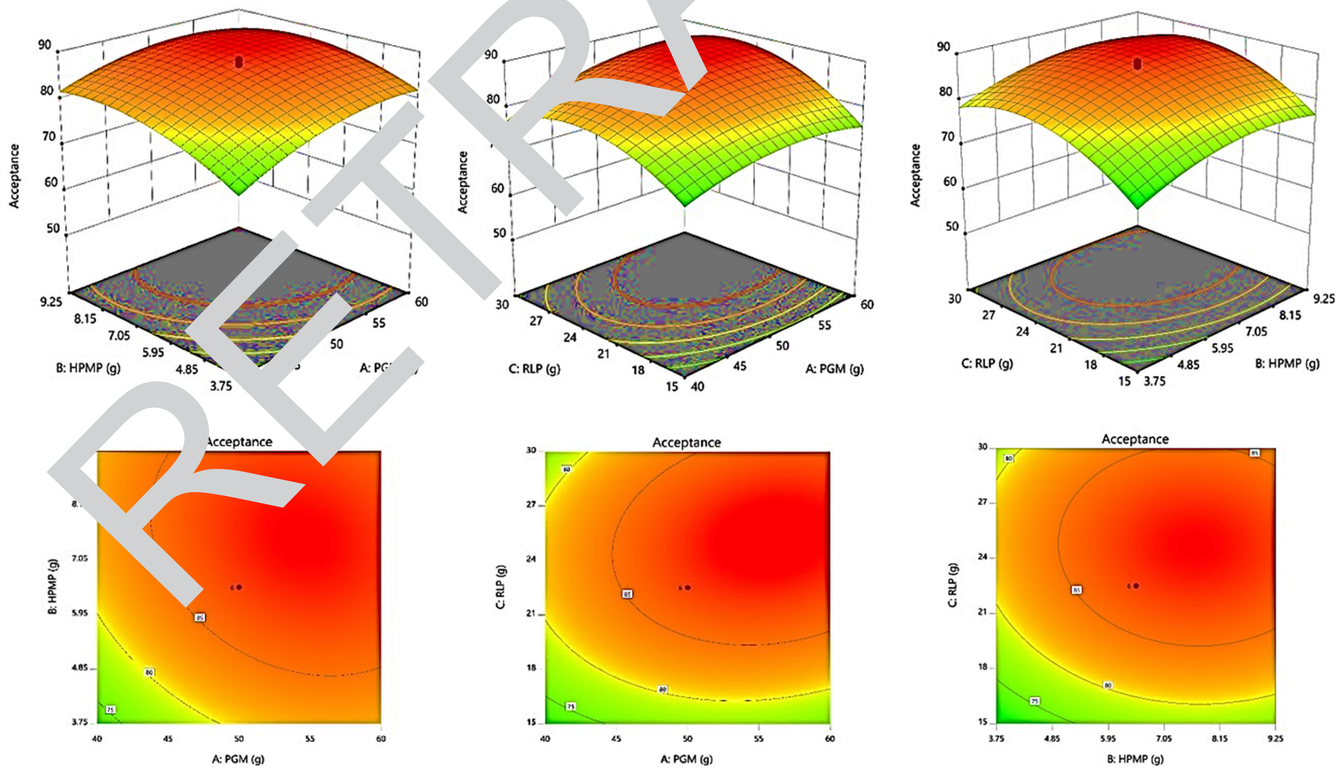

FIGURE 4 Response surface plots showing the interaction impacts of independent variables (PGM, HPMP, and RLP) on the sensory acceptance score of LS.

RETRACTED

RETRACTED

**TABLE 2** The level effects of major ingredients on peroxide value, aw, acidity, and total sensory scores of LS (linseed spread) out of maximum samples along with central composite design arrangement right after production

| | Coded values | | | Actual values | | | | | | |
|---|---|---|---|---|---|---|---|---|---|---|
| No. | PGM | HPMP | RLP | PGM (g) | HPMP (g) | RLP (g) | Peroxide value (meq $O_2$/kg) | Aw | Acidity (%OA)* | Acceptance sensory score (ASS)** |
| 1 | −1 | −1 | +1 | 40 | 3.75 | 30 | 1.38 | 0.50 | 0.216 | 71.4 |
| 2 | +1 | +1 | +1 | 60 | 9.25 | 30 | 1.30 | 0.65 | 0.22 | 82.3 |
| 3 | −1 | +1 | +1 | 40 | 9.25 | 30 | 1.40 | 0.51 | 0.22 | [illegible] |
| 4 | +1 | −1 | −1 | 60 | 3.75 | 15 | 0.74 | 0.76 | 0.159 | 72.1 |
| 5 | −1 | +1 | −14 | 40 | 9.25 | 15 | 0.97 | 0.65 | 0.172 | 63 |
| 6 | +1 | −1 | +1 | 60 | 3.75 | 30 | 1.21 | 0.65 | 0.209 | [illegible] |
| 7 | 0 | 0 | 0 | 50 | 6.5 | 22.5 | 1.02 | 0.63 | [illegible] | [illegible] |
| 8 | 0 | 0 | −2 | 50 | 6.5 | 7.5 | 0.87 | 0.71 | [illegible] | 48.3 |
| **9** | **0** | **0** | **0** | **50** | **6.5** | **22.5** | **1.02** | [illegible] | [illegible] | [illegible] |
| 10 | 0 | 0 | 0 | 50 | 6.5 | 22.5 | 1.04 | [illegible] | 0.183 | [illegible] |
| 11 | 0 | 0 | 0 | 50 | 6.5 | 22.5 | 1.03 | [illegible] | 0.18 | 89.2 |
| 12 | 0 | −2 | 0 | 50 | 1 | 22.5 | 1.01 | 0.68 | 0.184 | 82 |
| 13 | −2 | 0 | 0 | 30 | 6.5 | 22.5 | 1.12 | 0.53 | [illegible] | 51.8 |
| 14 | −1 | −1 | −1 | 40 | 3.75 | 15 | [illegible] | 0.64 | 0.174 | 60.2 |
| 15 | 0 | 0 | 0 | 50 | 6.5 | 22.5 | [illegible] | [illegible] | 0.181 | 89.1 |
| 16 | 0 | +2 | 0 | 50 | 12 | 22.5 | [illegible] | [illegible] | 0.192 | 87.8 |
| 17 | +1 | +1 | −1 | 60 | 9.25 | 15 | 0.85 | [illegible] | 0.165 | 84.1 |
| 18 | 0 | 0 | +2 | 50 | 6.5 | [illegible] | [illegible] | 0.46 | 0.258 | 59.3 |
| 19 | 0 | 0 | 0 | 50 | 6.5 | [illegible] | [illegible] | 0.62 | 0.18 | 91.2 |
| 20 | +2 | 0 | 0 | 70 | 6.5 | 22.5 | 0.85 | 0.78 | 0.187 | 79.5 |

* Oleic acid, ** Best LS sample with the highest sensory score obtained by using CDC and RSM and choosing the optimized levels of GMP, HPMP, and RLP considering one level lower and one level upper for each ingredient.

**TABLE 3** Regression coefficients and ANOVA of quadratic model for linseed spread

| | Responses | | | |
|---|---|---|---|---|
| Factor | Peroxide value (meq $O_2$/kg) | Water activity $a_w$ | Acidity (% OA) | Acceptance (score) |
| Intercept | [illegible] | 0.6252 | 0.1805 | 89.48 |
| PGM | −0.0706 | 0.0631 | −0.0028 | 6.60 |
| HPMP | 0.029 | 0.0019 | 0.0022 | 1.94 |
| RLP | [illegible] | −0.0619 | 0.0238 | 2.60 |
| (PGM) (HPMP) | 0.0188 | −0.0038 | 0.0019 | 2.20 |
| (PGM) (RLP) | 0.0063 | 0.0087 | 0.0019 | −1.97 |
| (HPMP) (RLP) | −0.0037 | 0.0012 | 0.0014 | −1.27 |
| $(PGM)^2$ | −0.011 | 0.0076 | 0.0025 | −6.12 |
| $(HPMP)^2$ | 0.009 | 0.0151 | 0.0017 | −1.27 |
| $(RLP)^2$ | 0.074 | −0.0099 | 0.0076 | −9.09 |
| Model F-value | 2033.84 (Sig.) | 192.89 (Sig.) | 811.21 (Sig.) | 153.47 (Sig.) |
| Lack of Fit F-value | 1.06 (Not Sig.) | 0.4069 (Not Sig.) | 0.2538 (Not Sig.) | 0.9326 (Not Sig.) |
| $R^2$ | 0.9995 | 0.9986 | 0.9967 | 0.9928 |
| Adj $R^2$ | 0.999 | 0.9974 | 0.9938 | 0.9863 |
| Pre R2 | 0.9973 | 0.9963 | 0.9828 | 0.9666 |
| Adequate Precision | 190.6144 | 104.3536 | 73.3292 | 36.7261 |

**TABLE 4** The practical ranges and appropriate levels of three independent variables for making LS with highest sensory score and permissible chemical indexes (PV, $a_w$, and acidity) along with comparison between the predicted and actual sensory scores

| | Acceptable ranges | Experimental values for optimized LS sample | Predicted value | Error (%) |
|---|---|---|---|---|
| PGM (g) | 40–60 | 50 | 48.053[a] | 3.89 |
| HPMP (g) | 3.75–9.25 | 6.5 | 6.277[a] | 4.20 |
| RLP (g) | 15–30 | 22.5 | 21.455[a] | 4.64 |
| Peroxide value (meq $O_2$/kg) | 0.74–1.78 | 1.02 | 1.023[b] | 0.29 |
| aw | 0.54–0.88 | 0.70 | 0.682[b] | 2.57 |
| Acidity (% oleic acid) | 0.159–258 | 0.182 | 0.179[b] | 1.64 |
| Acceptance (sensory score) | 50–88.3 | 88.3 | 84.893[b] | 3.85 |

aThe theoretical values of PGM, HPMP, and RLP numbers were gained when the actual PV, aw, acidity, and acceptance sensory score (obtained by experiment) inserted in equations of 7–10.

bThe three chemical indexes and acceptance sensory score were obtained when the appropriate levels of PGM, HPMP, and RLP inserted in equations of 7–10.

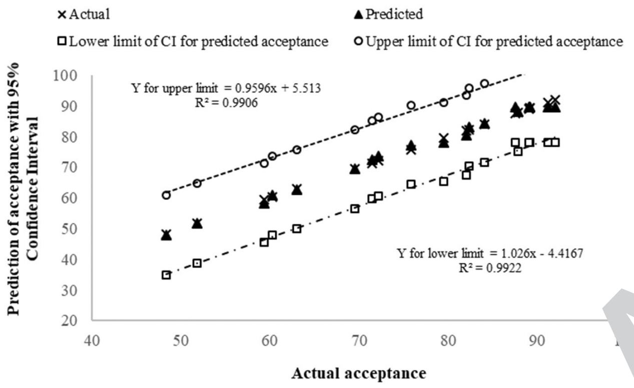

**FIGURE 5** The predicted linseed spread sensor[illegible]cores (obtained from a regression model) versus actu[illegible]ensory sc[illegible]es along with 95% upper and lower confidence i[illegible]vals for [illegible] predicted scores.

Table 3). Equation 7 also showed [illegible]at (RW[illegible])[illegible] and (HPMP)$^2$ [illegible]ad more impacts than (PGM)$^2$ on in[illegible]asing peroxid[illegible]lue. While (RWP)$^2$ and (HPMP)$^2$ had positi[illegible] coefficients, the coe[illegible]ient of (PGM)$^2$ was negative.

On the contrary, [illegible] inter[illegible]pt values for model of water activity (Equation 8[illegible] [illegible]nfirme[illegible]e increasi[illegible] and decreasing effects of, respective[illegible] two i[illegible]edients [illegible]GM[illegible]d HPMP) and one ingredient of RW[illegible]n $a_w$ (see [illegible]able 3). Equ[illegible]ion 8 also showed that (HPMP)$^2$ and (P[illegible])$^2$ had [illegible]than (RWP)$^2$ on increasing $a_w$. While (PGM)$^2$ an[illegible]PMP)$^2$ had positive coefficients, the coefficient of (RWP)$^2$ is nega[illegible].

Furthermore, [illegible]uation 9 showed that PGM, HPMP, and RWP had a significant impact on the acidity of the final WS. Additionally, the acceptance score of the WS was significantly influenced by the percentage of its three major ingredients, as Equation 10 shows this matter clearly (Table 4).

Equation 10 and Figure 5 display the high correlation between walnut spread sensory acceptance and the process variables in the selected model. This model was able to predict the total sensory score of linseeds spread with negligible error (from its actual number) using the selected process parameters.

### 4.4 | Optimi[illegible]ion the le[illegible]s of i[illegible]ependent and dependent v[illegible]a[illegible]s for long s[illegible]ge

RSM met[illegible] used to obta[illegible]itted regression model and find the opti[illegible]m values of PGM, HPM[illegible], and RWP for making the walnut spr[illegible]d with high q[illegible]lity and sensory scores as shown in Table 5. In [illegible]M, the desirab[illegible]y function is widely used to determine best leve[illegible] [illegible] combining [illegible]ree major independent variables and provide the most [illegible] responses (global suitability) in the final walnut [illegible]d. To define the desired level for each ingredient and its result[illegible] resp[illegible]e, the software suggests one solution with the highest attractiveness. The scale of the desirability function used for this purpose ranged between 0 (completely undesirable response) and [illegible] (fully desired response), and it was corresponding to the meaningful quality levels including: very good (1.0–0.8); good (0.8–0.63); fair (0.63–0.37); poor (0.37–0.2); and very poor (0.2–0). The preferred goal for desirable product was set within the ranges used for each independent variable. Simultaneously, it was our aim to produce WS with the minimum values of PV, $a_w$, and acidity, and maximum sensory acceptance score. The desirability 67.6% was obtained when the optimum levels of PGM, HPMP, and RWP were 47.402, 7.176, and 29.385 g, respectively. The maximum value of 67.6% means that the suggested proportion of the three major ingredients had good potential to produce an attractive walnut spread for consumers. As Table 5 shows, a small percentage error (<10%) was notified between the experimental and predicted values for the dependent variables. In other words, verification experiments demonstrated the adequacy of responses for the final product.

Beside the discussed dependent variables (PV, aw, and acidity), other chemical parameters of WS including the fiber, protein, fat, and ash contents along with pH can influence the sensory attributes and textural behavior of final product. Table 6 shows the results of chemical analyses performed in this study after 90 days of storage (see Table 6). The PV and acidity of the optimized sample stored for 90 days at 4°C, increased, respectively, from 0.72 to 1.07 meq $O_2$/kg and 0.09% to 0.196% mainly because it had low content of high-quality fat. However, the PV and acidity of hazelnut-cocoa spread stored for 90 days at similar conditions

RETRACTED

RETRACTED

TABLE 5 Chemical properties of optimized linseed spread

| Storage time | Factor | | | | | | | |
|---|---|---|---|---|---|---|---|---|
| | Fiber (%) | Protein (%) | Fat (%) | Ash (%) | pH | Aw | Acidity (%OA) | Peroxide value (meq $O_2$/kg) |
| 1st Day | 5.17 ±0.35[a] | 12.45 ±1.14[a] | 9.52 ±1.28[a] | 2.32 ±0.92[a] | 6.12 ±0.12[a] | 0.62 ±0.09[a] | 0.182 ±0.03[d] | 1.02 ±0.12[c] |
| 30th Day | 5.12 ±0.22[a] | 12.42 ±1.21[a] | 9.50 ±1.47[a] | 2.27 ±0.87[a] | 5.72 ±0.18[b] | 0.61 ±0.08[a] | 0.190 ±0.02[c] | 1.12 ±0.10[b] |
| 60th Day | 5.15 ±0.28[a] | 12.38 ±1.12[a] | 9.32 ±1.33[a] | 2.31 ±1.03[a] | 5.58 ±0.21[c] | 0.58 ±0.11[a] | 0.196 ±0.01[b] | 1.22 ±0.14[a] |
| 90th Day | 5.14 ±0.43[a] | 12.43 ±1.19[a] | 9.55 ±1.25[a] | 2.34 ±0.69[a] | 5.39 ±0.15[cd] | 0.56 ±0.09[a] | 0.199 ±0[illegible] | 1.[illegible]±0.17[a] |

*Note*: Data are means ± SD.
*For each column, means with superscripts (a–d) are significantly different ($p < .05$).

TABLE 6 Texture parameters of optimized linseed spread on 1st day of production

| Temperature | Hardness (N) | Adhesiveness (mJ) | Cohesiveness | Springiness | Gum[illegible]ness (N) | Chewiness (N) |
|---|---|---|---|---|---|---|
| 4°C | 1.84 | 0.4 | 0.33 | 0.26 | [illegible] | 0.16 |
| 25°C | 0.92 | 0.3 | 0.32 | 0.24 | 0.27 | 0.06 |
| 45°C | 0.74 | 0.2 | 0.29 | 0.[illegible]1 | 0.25 | 0.05 |

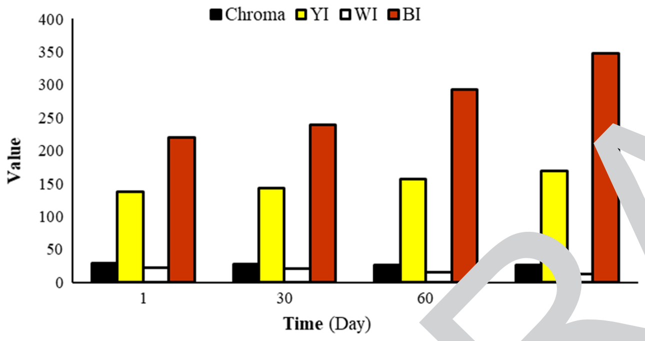

FIGURE 6 Effect of storage time o[illegible]olor paran[illegible]rs (WI, YI, BI, and chroma) of optimized linsee[illegible]ead at 4°C

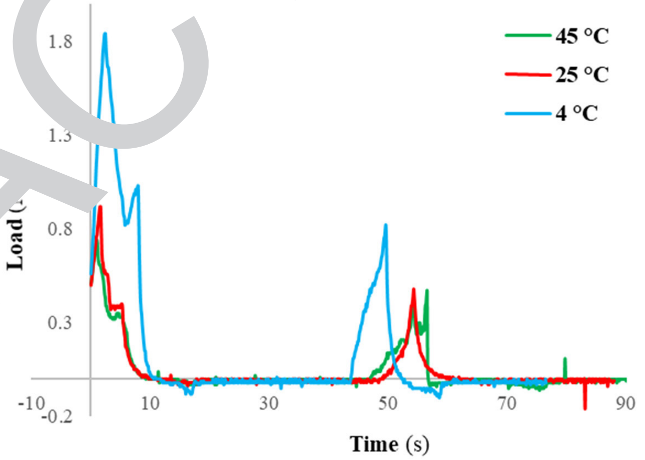

FIGURE 7 Effect of storing temperature on texture parameters of optimized linseed spread after production.

were much higher and, res[illegible]ctively, change[illegible]rom 4 to 6 meq $O_2$/kg and 0.71% to 0.77%[illegible]ainly because it ha[illegible]0% palm oil and 11% sunflower oil ([illegible]rakçi & Y[illegible]ırim, 2021). Although the protein content of some[illegible]ts ([illegible] as peanu[illegible]) spreads such as peanut spread reache[illegible] >20% [illegible]azaheri-Te[illegible]ani et al., 2009) and higher than WS[illegible]t our[illegible]ggestiv[illegible]pre[illegible] has considerable and unique health[illegible]enefits m[illegible]ly attribute[illegible] to its omega-3 fatty acids, fiber, and li[illegible]ns. A[illegible]onths' storage of WS did not make significan[illegible]nges on its protein (~13%), fat (~20%), and ash (~2%). Nevertheless, [illegible]s acidity and PV values increased significantly during this storag[illegible]time.

### 4.5 | Physical properties (color values and texture analysis)

In terms of customer thought and quality perception, the appealing features of hazelnut and chocolate spreads are crucial. Consumers are interested to accept spread products with a clear and shiny surface. Although walnut spread does not generate fat blooms appearance (which is a significant problem in chocolate products), the color values of L, a, and b play important roles on making final surface-color of walnut spread. While the obtained L value of WS was very close to chocolate spread ~30, it's a (redness to greenness) and b (yellowness to blueness) values, respectively, were higher and lower than chocolate spread. Almeida and Lannes (2017) reported that the L, a, and b values of the chocolate spread are ~30, 12, and 22, respectively. Most probably, higher a value of WS was due to the usage of Persian grape molasses, which has a dark red color (Almeida & Lannes, 2017).

Figure 6 shows the variations of color parameters, including chroma, whitish (WI), yellowness (YI), and brownness (BI) indexes of the WS samples during 90-day storage. While the BI and YI parameters of WS were increased as days of storage was extended (due to the browning and oxidation processes), its Chroma and WI reduced and made a product with a darker color. Researchers believe some complicated chemical processes involving sugars, acids, and phenolic compounds happen during storage even in cold (1–5°C) temperatures, and these reactions may reduce the astringency and bitterness during storage, resulting in a more stable color, pleasant smell, and superior flavor in different (nuts and chocolate) spreads (Baptista et al., 2021; Končarević et al., 2018). Storage conditions (mainly temperature and relative humidity) significantly altered the scales of color values and parameters (Nightingale et al., 2011). While storage of dark and milk chocolate in various conditions for 5 weeks reduce the visual and textural quality of chocolate (because of sugar or fat bloom formation [Andrae-Nightingale et al., 2009]), the suggestive walnut spread does not involve with these problems and defects.

Figure 7 represent the behavior-profile of walnut spread after a double compression test was performed at 4, 25, and 45°C. The analysis showed that the hardness (the peak force measured in 10 s as the first compression cycle) of optimized sample stored at 4, 25, and 45°C were 3.43, 2.76, and 2.16 N, respectively. The adhesiveness or the negative force area (happened after first bite ~15 s) was very small for each sample (regardless of its storage temperature) and ≤−0.2 N. In fact, this force was necessary to pull the compression probe away from each sample. The gumminess is related to the force needed to disintegrate each sample in the mouth (until it is ready to swallow). Again, the gumminess of [illegible] nut spread at 4°C (0.81 N) was much higher than similar ones k[illegible] at 25 and 45°C.

The springiness measures a force degree for ret[illegible] the WS product to its original size/shape after partial co[illegible]ession [illegible]ithout failure) applied between the tongue and teet[illegible]palate) for [illegible]eaching to a suitable consistency. This definition [illegible] food with higher elasticity values need higher [illegible]ding ener[illegible] By contrast, chewiness measures the time it [illegible] to chew a sa[illegible]e until it reaches a consistency that is su[illegible]able for [illegible]allowing. Although the springiness values for the sa[illegible]le at 4°C (0.35[illegible]re more significant than for 25 and 45°C, th[illegible]inal chewiness was c[illegible]siderably lower, suggesting that the [illegible]duced fo[illegible]ulations were easier to chew at 25°C. This may be d[illegible]o th[illegible]presence of RWP, which caused an increase in visc[illegible]ity. On [illegible] contrary, [illegible]stication is a complicated process th[illegible] involv[illegible] a variet[illegible] m[illegible]hanical forces, and accurately measur[illegible]g chewin[illegible] may be d[illegible]cult. In contrast to previous research [illegible] choc[illegible] [illegible] inclusion of corn syrup and butter increased [illegible] viscosity and adhesive characteristics (Dagadkhair et al., 2018; Ka[illegible]asili et al., 2020), the optimized sample of walnut spread was found [illegible] be low-sticky (0.1–0.6 mJ). Storage condition significantly impacted instrumental hardness, cohesiveness, adhesiveness, springiness, gumminess, and chewiness.

### 4.6 | Rheological description

The storage modulus (G') shows the elastic response (or stored energy) of food and reflects its solid-state behavior. However, loss modulus (G") reveals the viscous response (or dissipated heat energy) of a food (such as spread) a[illegible] shows its liquid-state behavior (Mantihal et al., 201[illegible]). Furthermore, i[illegible]nal friction between the components (mol[illegible]ules and particles) of a m[illegible]ving fluid causes viscous behavior an[illegible] production of f[illegible]tional heat. This friction is always associated wit[illegible] the conversion [illegible] deformation energy to heat energy. When the [illegible] material is [illegible]leased, the unused stored energy is a driving force to [illegible]t into its original shape (Pajin et al., 2013).

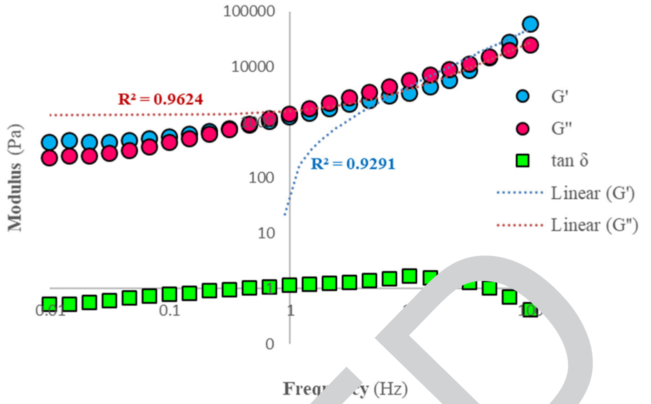

FIGURE 8 The effects of [illegible]equency ([illegible]plied for[illegible] on storage modulus (G'), loss (G") modu[illegible] and t[illegible] δ at 25°C in the linear viscoelasticity region [illegible]nseed [illegible]d. The uni[illegible] for both moduli are pressure or ene[illegible]/unit volum[illegible] LS aft[illegible] production.

[illegible]igure 8 illustrates the development of the storage (G') and loss ([illegible]) mo[illegible] because of linear range of viscoelasticity of WS versus applied frequency. As this figure shows at frequencies below 1 Hz, the storage-modulus values were considerably more than those in loss modulus (G' > G"). This is owing to the internal (chemical bonds connections, physiochemical) interactions between the molecules and particles of the materials in the resulting spread (Janmey & Schliwa, 2008). However, the situation of these two moduli was reversed when the frequency became higher than 1 Hz and subsequently, the loss modulus rose more than storage modulus.

Figure 8 also shows the loss tangent values (tan δ = G"/G') as a function of frequency. This is also known as the gel point or the sol/gel transition point and indicates that the sample moved from a liquid (or sol state) to a solid (or gel state) and vice versa during the viscoelastic measurement at different frequencies. When the tan δ > 1, the optimized walnut spread behaves like a viscus, whereas tan δ < 1 it behaves like an elastic (Manasi, 2019; Qaiser et al., 2021). These findings can be explained by the fact that the RWP plays a dual role in the formulation of WS and acts as a filler in the complicated network of the resulting spread. In other words, significant interactions take place between crystallized HPMP and PGM, and their outcomes make hydrodynamic polymers with the oil base of walnut paste. Researchers believe that low total elasticity of spreads is related to their high particle concentrations (Taylor et al., 2009).

Lecithin has been used in nuts (and specifically chocolate) spreads to control their viscoelasticity in the confectionery industry. This is the reason that the cocoa butter has been replaced with lecithin because lecithin has strong emulsification power and is much less expensive than cocoa butter. Moreover, usage of soy lecithin

RETRACTED

at 0.1%–0.3% level in every spread can reduce the same viscosity as over 10 times this amount of cocoa butter (Karnjanolarn & McCarthy, 2006). However, if the level of lecithin exceeds 0.3%–0.5%, unsuitable thickening of the chocolate occurs (Karnjanolarn & McCarthy, 2006). This is the reason we used lecithin below 0.5% to control the rheology of the final WS.

### 5 | CONCLUSION

A RSM design was used to combine different levels of RWP (roasted walnut paste), PGM (Persian grape molasses), and HPMP (high protein milk powder) and develop a valuable walnut spread. Consumer acceptability, $a_w$, acidity, and PV (peroxide value) were all determined to create a prediction model. To select the best areas, predictive algorithms were employed to create contour maps. Overlapping areas aided in the development of a walnut spread with optimized (high quality) compositions (containing 30 g RWP, 7.5 g HPMP, and 50 g PGM). The optimized walnut spread has a good potential to combine it with cocoa powder and enhance its consumer acceptance attributes (color and texture) specially for the international markets.

### ACKNOWLEDGMENT

We hereby thank the services of the laboratory of food eme[illegible] technologies and rheology of Tehran and Tabriz universities.

### ORCID

*Hamid Tavakolipour* https://orcid.org/0000-00[illegible]-0188[illegible]39

## References

AACC. (2000). *American Association of Ce[illegible] Chemists, [illegible]roved methods of the AACC* (10th ed.). American [illegible]ociation of Cer[illegible]Chemists.

Acan, B. G., Kilicli, M., Bursa, K., [illegible]ker, [illegible]S., Palabiyik, I., [illegible]lcu, M., Yaman, M., Gunes, R., & Konar, N. (202[illegible] Effect of grape pomace usage in chocolate spr[illegible] formulation on [illegible]ural, rheological and digestibility propert[illegible]. *LWT*, *138*, 110451.

Almeida, P. F., & Lanne[illegible]. C. D. S. [illegible]17). Effects of chicken by-product gelatin on the p[illegible]icochem[illegible]l properties and texture of chocolate spread. *Journal of [illegible]tudies*, *48*([illegible]92–402.

Amevor, P. M[illegible]a, D., [illegible]rimah, J. [illegible]018). Sensory evaluation, nutrien[illegible]ompos[illegible]on and [illegible]rob[illegible] load of cashew nut–chocolate s[illegible]ad. *Cogen[illegible]ood and Agri[illegible]ture*, *4*(1), 1480180.

An, J. [illegible] Kim, E[illegible] Lee, H. J., & Hahm, K. B. (2020). Dietary wa[illegible]od factor to [illegible]escue from NSAID-induced gastrointestinal mu[illegible]al damages. *Archives of Biochemistry and Biophysics*, *689*, 108466.

Andrae-Nightinga[illegible]L. M., Lee, S. Y., & Engeseth, N. J. (2009). Textural changes in chocolate characterized by instrumental and sensory techniques. *Journal of Texture Studies*, *40*(4), 427–444.

AOAC. (2000). *Official methods of analysis of the Association of Official Analytical Chemists*. AOAC International.

AOCS. (1989). *Official methods and recommended practices of the American oil Chemists'Society* (4th ed.). American Oil Chemists' Society.

Azizi Tabrizzad, M. H. (2020). Substitution of grape syrup instead of sugar for sponge cake production and its effect on firmness, volume, density and porosity. *Food Science and Technology*, *17*(105), 161–168.

Baptista, I., Valentin, D., Saldaña, E., & Behrens, J. (2021). Effects of packaging color on expected flavor, texture, and liking of chocolate in Brazil and France. *International Journal of Gastronomy and Food Science*, *24*, 100340.

Bolenz, S., Holm, M., & Langkrär, C. (2014). Improving particle size distribution and flow properties of milk chocolate produced by ball mill and blending. *European Food Research and Technology*, *238*(1), 139–147.

Coutinho, N. M., Silveira, M. R., Fernandes, L. M., Moraes, J., Pimentel, T. C., Freitas, M. Q., … Cruz, A. G. (2019). Processing chocolate milk drink by low-pressure cold plasma technology. *Food Chemistry*, *278*, 276–283.

Crawford, C. R., & Running, C. A. (2020). Additi[illegible]colate milk to diet corresponds to protein concentratio[illegible]hanges in [illegible]an saliva. *Physiology and Behavior*, *225*, 113080[illegible]

Dagadkhair, A. C., Andhale, R. R., Bhavsar, [illegible] & Pakhare, K. [illegible] (2018). Standardization for developm[illegible] of Mu[illegible]hocolate ba[illegible] by the utilization of banana pulp [illegible]der. *Journal [illegible]harma[illegible]gnosy and Phytochemistry*, *7*(3), 272[illegible]732.

dos Santos, C. S., Kanup, [illegible], Albuqu[illegible]que, M. A. C., Bedani, R., de Souza, C. H. B., Gi[illegible]elli, [illegible] Sa[illegible], S. M. I., & [illegible]act, J. N. R. (2020). Effect of enzym[illegible]c interes[illegible]ication on [illegible]e textural and nutritional prope[illegible]s of a probioti[illegible]ble [illegible]ead containing milk fat. *LWT*, *124*[illegible]91[illegible]

Ekrami, M., E[illegible]am-Djom[illegible]Z., Ghoreishy, S. A., Najari, Z., & Shakoury, N. (2019). Characterizatio[illegible]f a high-performance edible film based on [illegible]cilage functiona[illegible]d with pennyroyal (Mentha pulegium). *International Journal of Biological Macromolecules*, *133*, 529–537.

Fa[illegible]ri, F., Desogus, [illegible] Scano, E. A., Carboni, G., & Grosso, M. (2020). The effect of th[illegible]relative amount of ingredients on the rheological [illegible]roperties of se[illegible]olina doughs. *Sustainability*, *12*(7), 2705.

FAOS[illegible] (2019)[illegible]walnut (in shell) production in 2019, crops/regions/ worl[illegible]production quantity (pick lists)*. UN Food and Agriculture Organization.

[illegible]ermis[illegible], Z., Delcour, J. A., Deleu, L. J., & Manley, M. (2020). Characterization of white flour produced from roasted wheats differing in hardness and protein content. *Cereal Chemistry*, *97*(2), 339–348.

ISO. (2007). *Sensory analysis—General guidance for the design of test rooms*. Standard method of 8589.

Janmey, P. A., & Schliwa, M. (2008). Rheology. *Current Biology: CB*, *18*(15), R639–R641.

Karavasili, C., Gkaragkounis, A., Moschakis, T., Ritzoulis, C., & Fatouros, D. G. (2020). Pediatric-friendly chocolate-based dosage forms for the oral administration of both hydrophilic and lipophilic drugs fabricated with extrusion-based 3D printing. *European Journal of Pharmaceutical Sciences*, *147*, 105291.

Karnjanolarn, R., & McCarthy, K. L. (2006). Rheology of different formulations of milk chocolate and the effect on coating thickness. *Journal of Texture Studies*, *37*(6), 668–680.

Lončarević, I., Pajin, B., Fišteš, A., Šaponjac, V. T., Petrović, J., Jovanović, P., Vulić, J., & Zarić, D. (2018). Enrichment of white chocolate with blackberry juice encapsulate: Impact on physical properties, sensory characteristics and polyphenol content. *LWT*, *92*, 458–464.

Mahato, D. K., Keast, R., Liem, D. G., Russell, C. G., Cicerale, S., & Gamlath, S. (2021). Optimisation of natural sweeteners for sugar reduction in chocolate flavoured milk and their impact on sensory attributes. *International Dairy Journal*, *115*, 104922.

Manasi, I. (2019). *Structural basis for the rheology of molten chocolate: A multi-technique approach*. University of Edinburgh.

Mantihal, S., Prakash, S., Godoi, F. C., & Bhandari, B. (2019). Effect of additives on thermal, rheological and tribological properties of 3D printed dark chocolate. *Food Research International*, *119*, 161–169.

Mazaheri-Tehrani, M., Yeganehzad, S., Razmkhah-sharabiani, S., & Amjadi, H. (2009). Physicochemical and sensory properties of peanut spreads fortified with soy flour. *World Applied Sciences Journal*, *7*(2), 192–196.

Monteiro, S. H., Silva, E. K., Alvarenga, V. O., Moraes, J., Freitas, M. Q., Silva, M. C., … Cruz, A. G. (2018). Effects of ultrasound energy

RETRACTED

density on the non-thermal pasteurization of chocolate milk beverage. *Ultrasonics Sonochemistry*, *42*, 1–10.

Nightingale, L. M., Lee, S. Y., & Engeseth, N. J. (2011). Impact of storage on dark chocolate: Texture and polymorphic changes. *Journal of Food Science*, *76*(1), C142–C153.

Pajin, B., Dokić, L., Zarić, D., Šoronja-Simović, D., Lončarević, I., & Nikolić, I. (2013). Crystallization and rheological properties of soya milk chocolate produced in a ball mill. *Journal of Food Engineering*, *114*(1), 70–74.

Qaiser, A. A., Nazar, R., Anjum, M., Saeed, A., Zeeshan, M., Tahir, B., Muzaffar, M., & Jameel, N. (2021). Effects of composition, temperature and shear rate on chocolate milk rheology: An empirical modeling approach incorporating yield behavior. *International Journal of Food Engineering*, *17*, 561–569.

Savitri, D. A., Herlina, H., & Novijanto, N. (2021). Financial feasibility analysis of chocolate spread with coconut ingredients as Agroindustrial product. *Journal La Bisecoman*, *2*(2), 14–24.

Şen, S. M., & Karadeniz, T. (2015). The nutritional value of walnut. *Journal of Hygienic Engineering and Design*, *11*(18), 68–71.

Tarakçi, Z., & Yildirim, M. (2021). Effects of ghee and olive oil usage on cocoa hazelnut spreads during storage. *Journal of Food Processing and Preservation*, *45*(3), e15246.

Tavakolipour, H., Mokhtarian, M., & Kalbasi-Ashtari, A. (2020). Rheological modeling and activation energy of Persian grape molasses. *Journal of Food Process Engineering*, *43*(12), e13547.

Taylor, J., Van Damme, I., Johns, M., Routh, A., & Wilson, D. (2009). Shear rheology of molten crumb chocolate. *Journal of Food Science*, *74*(2), E55–E61.

Tobolková, B., Takáč, P., Mangová, B., & Kozánek, M. (2021). A comparative study of colour characteristics of thermally/non-thermally treated mealworm larvae (Tenebrio molitor) by means of UV/V is spectroscopy and multivariate analysis. *Journal of Food Measurement and Characterization*, *15*, 1–9.

Turner, T., & McNiven, M. (2011). In vitro N degradability and N digestibility of raw, roasted or extruded canola, linseed and soybean. *Agricultural and Food Science*, *20*(4), 298–30[illegible]

**How to cite this article:** Rahb[illegible] S., Tavakolip[illegible] H., & Kalbasi-Ashtari, A. (2023) [illegible]ects of m[illegible]or ingre[illegible] grape syrup, milk powder, and [illegible]nut pas[illegible] levels on physiochemical, rh[illegible]ogical, [illegible]ensory attr[illegible]utes of walnut spread. *Food Sc[illegible]ce & Nutrition*, [illegible] 4340[illegible]352. https://doi.org/10.100[illegible]sn3[illegible]07

RETRACTED
